# Remarks, Gleanings, and Extracts

**Published:** 1874-03

**Authors:** T. Curtis Smith

**Affiliations:** Ohio


### Remarks, Gleanings and Extracts.

BY T. CURTIS SMITH, M.D., OF OHIO.

*Deep Injection of Chloroform in TicDolereaux.*—Dr. J. B. Mattison, of Chester, N. J., reports to the Meigs and Mason Academy of Medicine, Feb. 19th, a case of tic dolereaux in which various agents had been used with very trifling benefit. The man had in former years been troubled with a trigeminal neuralgia, and afterwards with sciatica, which had continued for many years, during which time he had acquired the habit of using morphia hypodermically and in large quantities. This however afforded no relief from the facial pain.

December 22, 1873. One-third drachm of chloroform was injected near the infraorbital foramen by passing the needle beneath the upper lip, and carrying the point to near the emergence of the nerve, where the chloroform was discharged. A severe paroxysm followed its use, and several paroxysms occurred during several days following, but much lighter and far less frequent. After the 27th, no recurrence of the paroxysm took place. During this time he was placed on good general tonics, and also received three times a day hypodermically $\frac{1}{48}$ gr. strich. sulp. and $\frac{1}{60}$ gr. atropia sulp., which was continued for some time. No morphia being used after the 22d inst. In three weeks all desire for the latter agent had been dissipated. Six weeks later the man had entirely recovered his health and returned to "his vocation."

(The deep injection of chloroform seems likely to result in great benefit in this class of cases. Prof. Bartholow, who I believe was the originator of this method of treatment, relates three cases, in all of which it afforded relief, and that too, after prolonged trial with other agents accounted valuable, had failed. It occurs to my mind that a similar application of this method would prove highly beneficial in sciatica, by injecting the chloroform deeply, and as near as possible to the origin of the nerve affected.—Ed.)

*Removal of a Sound Kidney in the Human Subject.*—Prof. Brandt, of Klansenburg, relates a case of this character in the

*Wisner Medizinische Wochenschrift.*—A healthy man aet. 25, was stabbed with a bread knife in the left hyprochondrium, slight hemorrhage followed, and, about three hours later, a fleshy looking tumor was expelled through the wound during a fit of coughing, attended with severe pain. A bystander replaced it, but it was soon expelled again by coughing. He was twenty-four hours later, admitted to the hospital, when Dr. Brandt, after careful examination concluded that the tumor consisted of the left kidney. Its surface, with the ureter was somewhat torn, from which escaped a fluid, which was at first yellowish and transparent, but afterwards somewhat turbid, reaction, alkaline, specific gravity 1042 to 1052, contained a large quantity of albumen and mucin, some hemoglobin, traces of urea and an abundance of alkalies and alkaline earths. The sediment consisted of pus and blood-corpuscles, masses of nuclei, muco-fibrinous clots and epithelieum from the calyces and pelvis of the kidney. Dr. B. decided that the organ was rendered useless, and that its retention would endanger life, and therefore removed it, by tying the pedicle of the tumor in two parts, by means of a ligature passed through the middle, and cut it away with a knife. This was done on June 7th, the patient leaving the hospital on the 23d, convalescent. No peritonitis or uræmic symptoms occurred during the progress of the case.

The quantities of urine excreted from the 8th to the 22d, gradually increased with slight variation from 923 to 1513, grammes, was acid throughout, with specific gravity 1,010 to 1,046, and of normal composition; at first it was of a reddish-yellow color, but afterwards a clear yellow. Dr. Brandt has seen him several times since the operation. He has no signs of heart disease, but complains of a sense of oppression and fatigue, especially on ascending stairs, and says he cannot work as well as before. He was suspected however of saying this to avoid military service.—*British Medical Journal,* Dec. 20, 1873. *Clinic, January* 10, 1874.

*Complete Occlusion of the Os Uteri Complicating Labor.*—Prof. S. S. Todd, in the *Kansas City Medical Journal for January,* 1874, relates a case of this character. After pointing out the fact that such a case is readily mistaken at first for one where obliquity of the uterine axis exists in which the os cannot be reached early in labor, but which nature usually corrects later without aid or with very little interference, he proceeds to give the history of the removal of a small polypus from the cervix uteri, the woman at the time being about two and a half months pregnant, though he did not suspect it as she had been nineteen years married without having ever conceived that she was cognizant of. The polypus was removed on the 24th of March, 1873, and the delivery occurred on the 10th of the subsequent October. During fifteen hours before, various attempts were made to find the os, by the end of which time the head had descended to the floor of the pelvis, and the uterine walls had become "as thin as paper, the unequal surface of the fœtal cranium being clearly discernable through the attenuated intervening structures. It was apparent that if the old opening was not found, nature would soon make a new one of an ugly kind. "Finally, after diligent search, the site of the os seemed indicated by a threadlike sensation imparted to the finger at one point. Having no instrument at hand, an opening was made through the thinned walls with the finger-nail, and gradually enlarged, and in a short time delivery was completed. The woman made a rapid and satisfactory recovery."

*New Test for Morphia.*—Mr. T. B. Graves, before the British Pharma'l Society, describes the following extremely sensitive test for morphia: The alkaloid must be first separated, from all extraneous matter. A minute portion of it is then heated with about six drops of pure sulphuric acid, to which is then added a very small quantity of pure perchlorate of potassium (perfectly free from chlorate.) The liquid surrounding the perchlorate immediately assumes a deep brown color, which soon extends over the greater part of the acid. Warming increases the delicacy of the test. No other alkaloid gives a similar reaction.—*Boston Journal Chem.*—*Detroit Rev.*

*Case of Paracentesis Throacis, Recovery.*—Prof. George Halley, in the *Kansas City Medical Journal*, for January, 1874, relates a very interesting case of this kind. A man had been attacked with pleuritis, in February, 1873, which confined him to his bed for a time. On resuming labor he found himself suffering from shortness of breath, which so increased as to cause him to cease labor, being also debilitated. He was evidently suffering from intrathoracic effusion, and after a consultation, was tapped by Dr. Paul Eve, of Nashville, the man's place of residence. For three days relief was had, but soon the difficulty returned, even worse than before, and he was advised to "go west." Arriving at Kansas City, June 1st, he was too ill to travel farther. Dr. Halley found two-thirds of the chest anasarcous reaching up high in the neck, and below to the crest of the illium. Pulse 134, and laboured, apex of heart being to right of the sternum." The abdominal swelling had been previously mistaken for an enlarged spleen, but it proved to be fluid in the pleuritic cavity that had crowded down the diaphragm before it. Dr. Halley "punctured the chest between the seventh and eighth ribs, in a line below the center of the axillary space," using a Davidson's syringe and lower half of a male catheter as an aspirator. He drew off "*fifteen pints of pus,*" the last being mixed with blood. From this date the patient improved rapidly, and the lung soon assumed its functions. He was placed on tonic and alterative treatment.

*Influence of Alcoholism on Conception.*—M. Auguste Voison, at a meeting of the French Med. Psych. Asso., made the following interesting statements: 1st. The influence of the various alcoholic drinks upon the form of mental derangement.

2d. On conception during drunkenness. As the result of seventeen cases which were fully examined, it would appear that wine, brandy and absinthe exercise an almost identical influence upon the products of conception. Epilepsy, convulsions in childhood, and chronic myelitis are the possible consequences of conception during drunkenness, whatever the intoxicating agent may be.

3d. On conception during chronic alcoholism without drukenness. In eighteen cases observed, there were born eight idiots and ten epileptics.

Of the eight idiots four were the issue of fathers who indulged in wine, two of brandy drinkers, and two had mothers that drank brandy.

Of the ten epileptics, five had fathers who drank brandy and wine, three who used wine only, and two who used absinthe.—*Clinic, Feb.* 7, 1874.

*Ergotin in Varix of Pregnancy.*—Dr. P. Ruge reports an interresting case of this character, in the *Berlina Klinische Wochenschrift,* Nov. 3, 1873. A woman aged 36, who, when a child, had been scrofulous and anemic, had suffered from large varices in her first pregnancy, during which, one of them had ruptured. She presented herself in the eighth month of the second gestation for treatment. The varices were upon the legs and posterior wall of the vagina, for the relief of which, ergotin was used hypodermically, on the left leg and thigh over the site largest ectasia. From 0.06-0.1 grm. was injected every 2 to 6 days. The effects of the first dose was quite visible, and after the seventh they had almost entirely disappeared on the left leg, but were still present in the right. The difference before and after treatment is shown by two drawings which exhibit the remarkable change effected. No abscess resulted from the use of the ergotin injections, though there was painful infiltration; nor had the remedy any effect in inciting uterine contractions, as labor did not occur till four weeks from the time of the first injection.—*Clinic, January* 16, 1874.

*Arsenic for the Relief of Asthma.*—Dr. Paul Coneyges, in the *American Journal Medical Sciences*, January, 1874, commends this agent as most valuable for the relief of this most distressing disease. It should be given until its physiological effects are plainly manifested, as prophylactic, which often necessitates its long continued use. He also commends it highly for the purpose of relieving the spasmodic dyspnœa of the different attacks. He says, "I have seen it relieve sufferers after all other recognized drugs have failed. Cases that did not yield in the beginning have done so by a continuation of its use, assisted by medicine to countereact indigestion, bronchitis, etc. I have an excessive dyspaœa, resulting from an aggravated attack of asthma, almost instantaneously relieved by the use of Fowler's Solution hypodermically. By instantaneous, I mean, with the same rapidity that a proper dose of morphia, similarly given, will relieve pain." Care should be taken not to overdose by this method, nor is it recommended to be used hypodermically until it has been tried fairly by the usual method.

*For Gastralgia.*—After clearing out the alimentary canal thoroughly, use ℞. agent nitr. grs. xx.; quinia sulp. ℈ii.; ext. hyoscyam ℈i.; morphia sulp. grs. iiss.; ol piper nigr. q. s.; M. ft. Pil xx. S. One after each meal. A permanent cure may be expected in cases where this is well received, however chronic the disease.

*Prevention of Mercurial Poisoning.*—It has been discovered by Jules Myer that operatives exposed to mercurial fumes escape wholly their disagreeable effects by keeping the atmosphere moderately charged with the vapor of ammonia.—*Detroit Review.*

---

